# Methyl 2-({6-[(1-meth­oxy-2-methyl-1-oxopropan-2-yl)carbamo­yl]pyridin-2-yl}formamido)-2-methyl­propano­ate

**DOI:** 10.1107/S1600536812014651

**Published:** 2012-04-13

**Authors:** Mohamed A. Al-Omar, Abdel-Galil E. Amr, Hazem A. Ghabbour, Ching Kheng Quah, Hoong-Kun Fun

**Affiliations:** a Pharmaceutical Chemistry Department, College of Pharmacy, King Saud University, Riyadh 11451, Saudi Arabia; b Drug Exploration & Development Chair (DEDC), College of Pharmacy, King Saud University, Riyadh 11451, Saudi Arabia; c Applied Organic Chemistry Department, National Research Center, Dokki 12622, Cairo, Egypt; d X-ray Crystallography Unit, School of Physics, Universiti Sains Malaysia, 11800 USM, Penang, Malaysia

## Abstract

In the title compound, C_17_H_23_N_3_O_6_, the two meth­oxy­carbonyl C—O—C=O planes are inclined at dihedral angles of 5.3 (4) and 83.9 (4)° with respect to the central pyridine ring. An intra­molecular N—H⋯O hydrogen bond generates an *S*(5) ring motif. In the crystal, mol­ecules are linked into a chain along the *c* axis *via* C—H⋯O hydrogen bonds.

## Related literature

For general background to and the pharmacological activity of the title compound, see: Abou-Ghalia & Amr (2004 ▶); Abou-Ghalia *et al.* (2003 ▶); Al-Omar & Amr (2010 ▶); Amr (2000 ▶); Attia *et al.* (1997 ▶, 2000 ▶); Amr *et al.* (2009 ▶); Fakhr *et al.* (2008 ▶). For standard bond-length data, see: Allen *et al.* (1987 ▶). For hydrogen-bond motifs, see: Bernstein *et al.* (1995 ▶).
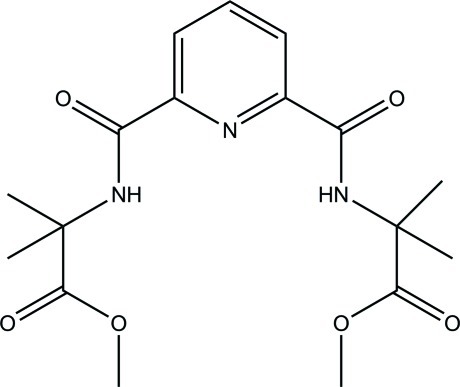


## Experimental

### 

#### Crystal data

C_17_H_23_N_3_O_6_
*M* *_r_* = 365.38
Orthorhombic, 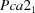

*a* = 10.2307 (10) Å
*b* = 9.3038 (11) Å
*c* = 20.652 (2) Å
*V* = 1965.8 (4) Å^3^
*Z* = 4
Cu *K*α radiation
μ = 0.79 mm^−1^
*T* = 296 K
0.63 × 0.52 × 0.09 mm

#### Data collection

Bruker SMART APEXII CCD area-detector diffractometer
Absorption correction: multi-scan (*SADABS*; Bruker, 2009 ▶) *T*_min_ = 0.560, *T*_max_ = 0.932
7477 measured reflections
1898 independent reflections
1249 reflections with *I* > 2σ(*I*)
*R*_int_ = 0.043

#### Refinement

*R*[*F* ^2^ > 2σ(*F* ^2^)] = 0.050
*wR*(*F* ^2^) = 0.133
*S* = 0.91
1898 reflections
250 parameters
1 restraint
H atoms treated by a mixture of independent and constrained refinement
Δρ_max_ = 0.19 e Å^−3^
Δρ_min_ = −0.17 e Å^−3^

### 

Data collection: *APEX2* (Bruker, 2009 ▶); cell refinement: *SAINT* (Bruker, 2009 ▶); data reduction: *SAINT*; program(s) used to solve structure: *SHELXTL* (Sheldrick, 2008 ▶); program(s) used to refine structure: *SHELXTL*; molecular graphics: *SHELXTL*; software used to prepare material for publication: *SHELXTL* and *PLATON* (Spek, 2009 ▶).

## Supplementary Material

Crystal structure: contains datablock(s) global, I. DOI: 10.1107/S1600536812014651/is5109sup1.cif

Structure factors: contains datablock(s) I. DOI: 10.1107/S1600536812014651/is5109Isup2.hkl

Supplementary material file. DOI: 10.1107/S1600536812014651/is5109Isup3.cml

Additional supplementary materials:  crystallographic information; 3D view; checkCIF report

## Figures and Tables

**Table 1:** Hydrogen-bond geometry (Å, °)

*D*—H⋯*A*	*D*—H	H⋯*A*	*D*⋯*A*	*D*—H⋯*A*
N2—H1*N*2⋯O2	0.85 (4)	2.08 (4)	2.614 (5)	120 (3)
C3—H3*A*⋯O6^i^	0.93	2.49	3.204 (6)	134

Symmetry code: (i) 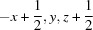.

**Fig. 1.**
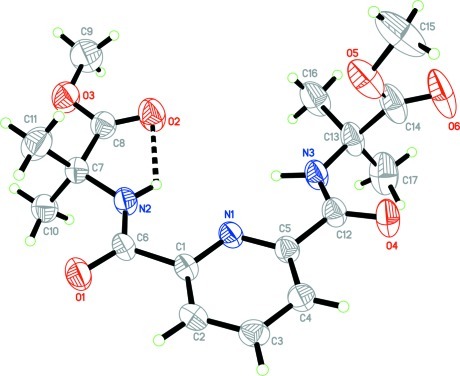
The molecular structure of the title compound showing 30% probability displacement ellipsoids for non-H atoms. The intramolecular hydrogen bond is shown as a dashed line.

**Fig. 2.**
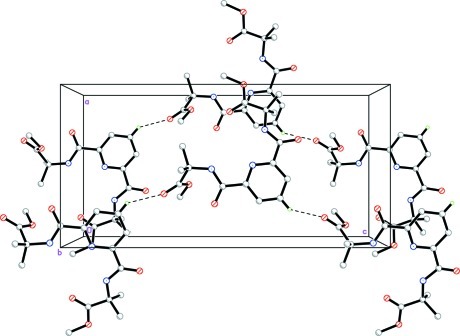
A crystal packing diagram of the title compound, viewed along the b axis. H atoms not involved in hydrogen bonds (dashed lines) have been omitted for clarity.

## supplementary crystallographic information

### Comment

In our previous work, we have reported that certain substituted pyridines and Schiff base derivatives are antimicrobial, anti-inflammatory and anticancer agents (Abou-Ghalia & Amr, 2004; Abou-Ghalia *et al.*, 2003; Al-Omar & Amr, 2010; Amr, 2000; Attia *et al.*, 1997, 2000). In continuation of our interests in the chemical and pharmacological properties of disubstituted pyridine derivatives (Amr *et al.*, 2009; Fakhr *et al.*, 2008), we report herein the synthesis and antimicrobial activities of the title compound.

In the title molecule (Fig. 1), the two methoxycarbonyl moieties (O2/O3/C8/C9 and O5/O6/C14/C15) are nearly planar [maximum deviations of 0.005 (5) and 0.009 (5) Å at atoms C8 and C14, respectively] and are inclined at angles of 5.3 (4) and 83.9 (4)° with the pyridine ring (N1/C1–C5). Bond lengths (Allen *et al.*, 1987) and angles are within normal ranges. The molecular structure is stabilized by an intramolecular N2—H1N2···O2 hydrogen bond (Table 1), which generates an *S*(5) ring motif (Bernstein *et al.*, 1995).

In the crystal (Fig. 2), molecules are linked into one-dimensional chains propagating along the [001] direction *via* intermolecular C3—H3A···O6 hydrogen bonds (Table 1).

### Experimental

To a solution of 2-methylalanine methyl esters (2 mmol), 2,6-pyridinedicarboyl dichloride (1 mmol) in dichloromethane (15 mL) was added at -10 °C with stirring. Triethylamine was added drop wise to the reaction mixture in order to keep the reaction mixture slightly basic (pH ~ 8). Stirring was continued for 3 h more at -15 °C and then 12 h at r.t. The reaction mixture was then washed with water, 1N hydrochloric acid, 1N sodium bicarbonate and finally with water and dried over anhydrous calcium chloride. The solvent was evaporated under reduced pressure to dryness and the obtained solid was crystallized from chloroform to give the titled bis-ester.

### Refinement

Atoms H1N2 and H1N3 were located in a difference Fourier map and refined freely [N—H = 0.84 (4) and 0.95 (5) Å]. The remaining hydrogen atoms were positioned geometrically (C—H = 0.93 or 0.96 Å) and were refined using a riding model, with *U*_iso_(H) = 1.2 or 1.5 *U*_eq_(C). A rotating group model was applied to the methyl groups. Even though Cu radiation was used, there was not enough anomalous dispersion to determine the absolute configuration. Thus, Friedel pairs have been merged.

### Crystal data

**Table:**

C_17_H_23_N_3_O_6_	*F*(000) = 776
*M**_r_* = 365.38	*D*_x_ = 1.235 Mg m^−^^3^
Orthorhombic, *P**c**a*2_1_	Cu *K*α radiation, λ = 1.54178 Å
Hall symbol: P 2c -2ac	Cell parameters from 874 reflections
*a* = 10.2307 (10) Å	θ = 4.8–49.5°
*b* = 9.3038 (11) Å	µ = 0.79 mm^−^^1^
*c* = 20.652 (2) Å	*T* = 296 K
*V* = 1965.8 (4) Å^3^	Plate, colourless
*Z* = 4	0.63 × 0.52 × 0.09 mm

### Data collection

**Table:**

Bruker SMART APEXII CCD area-detector diffractometer	1898 independent reflections
Radiation source: fine-focus sealed tube	1249 reflections with *I* > 2σ(*I*)
Graphite monochromator	*R*_int_ = 0.043
φ and ω scans	θ_max_ = 70.3°, θ_min_ = 6.4°
Absorption correction: multi-scan (*SADABS*; Bruker, 2009)	*h* = −12→10
*T*_min_ = 0.560, *T*_max_ = 0.932	*k* = −10→11
7477 measured reflections	*l* = −24→13

### Refinement

**Table:**

Refinement on *F*^2^	Secondary atom site location: difference Fourier map
Least-squares matrix: full	Hydrogen site location: inferred from neighbouring sites
*R*[*F*^2^ > 2σ(*F*^2^)] = 0.050	H atoms treated by a mixture of independent and constrained refinement
*wR*(*F*^2^) = 0.133	*w* = 1/[σ^2^(*F*_o_^2^) + (0.0914*P*)^2^] where *P* = (*F*_o_^2^ + 2*F*_c_^2^)/3
*S* = 0.91	(Δ/σ)_max_ = 0.001
1898 reflections	Δρ_max_ = 0.19 e Å^−^^3^
250 parameters	Δρ_min_ = −0.17 e Å^−^^3^
1 restraint	Extinction correction: *SHELXTL* (Sheldrick, 2008), Fc^*^=kFc[1+0.001xFc^2^λ^3^/sin(2θ)]^-1/4^
Primary atom site location: structure-invariant direct methods	Extinction coefficient: 0.0102 (11)

### Special details

**Table:**

Geometry. All esds (except the esd in the dihedral angle between two l.s. planes) are estimated using the full covariance matrix. The cell esds are taken into account individually in the estimation of esds in distances, angles and torsion angles; correlations between esds in cell parameters are only used when they are defined by crystal symmetry. An approximate (isotropic) treatment of cell esds is used for estimating esds involving l.s. planes.
Refinement. Refinement of F^2^ against ALL reflections. The weighted R-factor wR and goodness of fit S are based on F^2^, conventional R-factors R are based on F, with F set to zero for negative F^2^. The threshold expression of F^2^ > 2sigma(F^2^) is used only for calculating R-factors(gt) etc. and is not relevant to the choice of reflections for refinement. R-factors based on F^2^ are statistically about twice as large as those based on F, and R- factors based on ALL data will be even larger.

### Fractional atomic coordinates and isotropic or equivalent isotropic displacement parameters (Å^2^)

**Table:**

	*x*	*y*	*z*	*U*_iso_*/*U*_eq_	
O1	0.6692 (3)	0.7355 (4)	0.72873 (15)	0.1024 (10)	
O2	0.8492 (3)	0.6849 (5)	0.51295 (18)	0.1213 (13)	
O3	1.0304 (3)	0.5979 (4)	0.55776 (19)	0.1156 (12)	
O4	0.2658 (2)	0.9156 (4)	0.46691 (14)	0.1017 (11)	
O5	0.3806 (4)	0.6611 (7)	0.3829 (2)	0.1392 (17)	
O6	0.3250 (4)	0.8241 (7)	0.3084 (2)	0.170 (2)	
N1	0.5055 (3)	0.8326 (3)	0.58446 (14)	0.0676 (8)	
N2	0.7327 (3)	0.7306 (4)	0.62369 (18)	0.0745 (8)	
H1N2	0.715 (4)	0.744 (4)	0.584 (2)	0.068 (12)*	
N3	0.4811 (3)	0.8725 (5)	0.45674 (16)	0.0830 (10)	
H1N3	0.554 (5)	0.851 (5)	0.484 (3)	0.102 (14)*	
C1	0.5188 (3)	0.8156 (4)	0.64814 (17)	0.0665 (9)	
C2	0.4185 (4)	0.8403 (4)	0.69200 (19)	0.0740 (10)	
H2A	0.4315	0.8263	0.7361	0.089*	
C3	0.2992 (4)	0.8860 (5)	0.66876 (19)	0.0774 (10)	
H3A	0.2308	0.9055	0.6971	0.093*	
C4	0.2830 (3)	0.9021 (5)	0.60377 (18)	0.0737 (10)	
H4A	0.2027	0.9306	0.5870	0.088*	
C5	0.3875 (3)	0.8757 (4)	0.5630 (2)	0.0684 (9)	
C6	0.6502 (3)	0.7562 (5)	0.67077 (17)	0.0720 (9)	
C7	0.8613 (3)	0.6622 (4)	0.6287 (2)	0.0741 (9)	
C8	0.9097 (4)	0.6522 (5)	0.5605 (2)	0.0862 (11)	
C9	1.0870 (6)	0.5824 (10)	0.4936 (3)	0.147 (3)	
H9A	1.1591	0.5165	0.4954	0.220*	
H9B	1.1173	0.6742	0.4786	0.220*	
H9C	1.0219	0.5462	0.4643	0.220*	
C10	0.9549 (4)	0.7569 (6)	0.6675 (3)	0.1005 (15)	
H10A	0.9658	0.8474	0.6458	0.151*	
H10B	1.0381	0.7099	0.6712	0.151*	
H10C	0.9196	0.7731	0.7100	0.151*	
C11	0.8503 (6)	0.5137 (6)	0.6576 (3)	0.1151 (17)	
H11A	0.7937	0.4559	0.6313	0.173*	
H11B	0.8149	0.5205	0.7006	0.173*	
H11C	0.9353	0.4703	0.6595	0.173*	
C12	0.3733 (3)	0.8903 (5)	0.49148 (19)	0.0774 (11)	
C13	0.4893 (4)	0.8851 (7)	0.3870 (2)	0.0988 (17)	
C14	0.3863 (4)	0.7913 (10)	0.3552 (2)	0.121 (2)	
C15	0.2980 (11)	0.5560 (12)	0.3517 (4)	0.212 (5)	
H15A	0.3126	0.4633	0.3709	0.317*	
H15B	0.3185	0.5520	0.3064	0.317*	
H15C	0.2080	0.5827	0.3571	0.317*	
C16	0.6234 (4)	0.8267 (9)	0.3661 (3)	0.135 (3)	
H16A	0.6336	0.7301	0.3816	0.203*	
H16B	0.6912	0.8862	0.3838	0.203*	
H16C	0.6291	0.8273	0.3197	0.203*	
C17	0.4747 (6)	1.0409 (9)	0.3669 (3)	0.143 (3)	
H17A	0.4022	1.0830	0.3896	0.215*	
H17B	0.4594	1.0461	0.3211	0.215*	
H17C	0.5533	1.0924	0.3774	0.215*	

### Atomic displacement parameters (Å^2^)

**Table:**

	*U*^11^	*U*^22^	*U*^33^	*U*^12^	*U*^13^	*U*^23^
O1	0.101 (2)	0.140 (3)	0.0657 (19)	0.0175 (18)	−0.0122 (14)	0.0065 (18)
O2	0.096 (2)	0.188 (4)	0.080 (2)	0.027 (2)	0.0005 (17)	0.002 (2)
O3	0.0874 (19)	0.157 (3)	0.102 (3)	0.0322 (18)	0.0008 (16)	−0.005 (2)
O4	0.0631 (13)	0.167 (3)	0.0754 (17)	0.0001 (15)	−0.0090 (13)	0.0276 (19)
O5	0.133 (3)	0.200 (5)	0.085 (3)	−0.049 (3)	−0.009 (2)	−0.016 (3)
O6	0.114 (3)	0.318 (7)	0.078 (2)	−0.045 (4)	−0.038 (2)	0.024 (3)
N1	0.0623 (15)	0.085 (2)	0.0558 (18)	−0.0025 (14)	−0.0017 (11)	−0.0001 (14)
N2	0.0652 (15)	0.096 (2)	0.0620 (19)	0.0030 (14)	−0.0075 (14)	0.0038 (18)
N3	0.0638 (16)	0.130 (3)	0.0552 (18)	0.0040 (16)	−0.0019 (13)	0.0054 (17)
C1	0.0708 (19)	0.074 (2)	0.054 (2)	−0.0044 (15)	−0.0051 (14)	0.0036 (17)
C2	0.085 (2)	0.080 (2)	0.057 (2)	−0.0078 (18)	0.0009 (17)	−0.005 (2)
C3	0.071 (2)	0.090 (3)	0.071 (3)	−0.0034 (19)	0.0125 (17)	−0.008 (2)
C4	0.0646 (19)	0.088 (3)	0.068 (2)	−0.0023 (17)	0.0045 (15)	−0.0001 (19)
C5	0.0603 (17)	0.081 (2)	0.064 (2)	−0.0025 (15)	−0.0025 (15)	−0.0004 (18)
C6	0.075 (2)	0.087 (3)	0.054 (2)	−0.0019 (18)	−0.0104 (16)	0.0023 (18)
C7	0.0707 (19)	0.079 (2)	0.072 (2)	0.0061 (17)	−0.0080 (17)	0.001 (2)
C8	0.072 (2)	0.101 (3)	0.086 (3)	0.0066 (19)	−0.005 (2)	−0.004 (3)
C9	0.104 (4)	0.217 (8)	0.118 (4)	0.037 (4)	0.019 (3)	−0.021 (5)
C10	0.078 (2)	0.123 (4)	0.100 (3)	0.012 (2)	−0.020 (2)	−0.019 (3)
C11	0.125 (4)	0.100 (4)	0.121 (4)	0.025 (3)	0.010 (3)	0.021 (3)
C12	0.0661 (19)	0.105 (3)	0.061 (2)	−0.0039 (18)	−0.0013 (16)	0.008 (2)
C13	0.066 (2)	0.174 (5)	0.056 (2)	−0.004 (2)	−0.0016 (15)	0.014 (3)
C14	0.086 (3)	0.222 (7)	0.056 (3)	−0.027 (3)	−0.004 (2)	0.012 (4)
C15	0.227 (10)	0.274 (12)	0.134 (6)	−0.117 (9)	−0.011 (6)	−0.065 (7)
C16	0.076 (3)	0.249 (8)	0.081 (3)	−0.005 (3)	0.010 (2)	−0.032 (4)
C17	0.111 (4)	0.213 (8)	0.106 (4)	−0.015 (4)	0.003 (3)	0.066 (5)

### Geometric parameters (Å, º)

**Table:**

O1—C6	1.228 (5)	C7—C8	1.497 (6)
O2—C8	1.200 (6)	C7—C11	1.509 (7)
O3—C8	1.335 (5)	C7—C10	1.528 (6)
O3—C9	1.454 (7)	C9—H9A	0.9600
O4—C12	1.234 (4)	C9—H9B	0.9600
O5—C14	1.341 (9)	C9—H9C	0.9600
O5—C15	1.444 (8)	C10—H10A	0.9600
O6—C14	1.191 (6)	C10—H10B	0.9600
N1—C1	1.332 (5)	C10—H10C	0.9600
N1—C5	1.347 (4)	C11—H11A	0.9600
N2—C6	1.309 (5)	C11—H11B	0.9600
N2—C7	1.466 (5)	C11—H11C	0.9600
N2—H1N2	0.84 (4)	C13—C17	1.516 (9)
N3—C12	1.326 (5)	C13—C14	1.518 (8)
N3—C13	1.447 (6)	C13—C16	1.538 (7)
N3—H1N3	0.95 (5)	C15—H15A	0.9600
C1—C2	1.387 (5)	C15—H15B	0.9600
C1—C6	1.527 (5)	C15—H15C	0.9600
C2—C3	1.379 (6)	C16—H16A	0.9600
C2—H2A	0.9300	C16—H16B	0.9600
C3—C4	1.361 (6)	C16—H16C	0.9600
C3—H3A	0.9300	C17—H17A	0.9600
C4—C5	1.382 (5)	C17—H17B	0.9600
C4—H4A	0.9300	C17—H17C	0.9600
C5—C12	1.491 (6)		
			
C8—O3—C9	116.4 (4)	C7—C10—H10A	109.5
C14—O5—C15	116.6 (7)	C7—C10—H10B	109.5
C1—N1—C5	116.8 (3)	H10A—C10—H10B	109.5
C6—N2—C7	127.2 (4)	C7—C10—H10C	109.5
C6—N2—H1N2	123 (3)	H10A—C10—H10C	109.5
C7—N2—H1N2	109 (3)	H10B—C10—H10C	109.5
C12—N3—C13	125.2 (3)	C7—C11—H11A	109.5
C12—N3—H1N3	111 (3)	C7—C11—H11B	109.5
C13—N3—H1N3	123 (3)	H11A—C11—H11B	109.5
N1—C1—C2	123.4 (3)	C7—C11—H11C	109.5
N1—C1—C6	115.8 (3)	H11A—C11—H11C	109.5
C2—C1—C6	120.7 (3)	H11B—C11—H11C	109.5
C3—C2—C1	118.6 (4)	O4—C12—N3	122.9 (4)
C3—C2—H2A	120.7	O4—C12—C5	120.8 (3)
C1—C2—H2A	120.7	N3—C12—C5	116.3 (3)
C4—C3—C2	119.0 (4)	N3—C13—C17	110.1 (5)
C4—C3—H3A	120.5	N3—C13—C14	110.1 (4)
C2—C3—H3A	120.5	C17—C13—C14	111.3 (5)
C3—C4—C5	119.1 (4)	N3—C13—C16	107.6 (4)
C3—C4—H4A	120.4	C17—C13—C16	110.4 (5)
C5—C4—H4A	120.4	C14—C13—C16	107.2 (5)
N1—C5—C4	123.1 (4)	O6—C14—O5	123.7 (7)
N1—C5—C12	116.1 (3)	O6—C14—C13	124.6 (8)
C4—C5—C12	120.8 (3)	O5—C14—C13	111.4 (4)
O1—C6—N2	126.4 (4)	O5—C15—H15A	109.5
O1—C6—C1	119.7 (4)	O5—C15—H15B	109.5
N2—C6—C1	114.0 (3)	H15A—C15—H15B	109.5
N2—C7—C8	104.9 (3)	O5—C15—H15C	109.5
N2—C7—C11	111.0 (4)	H15A—C15—H15C	109.5
C8—C7—C11	109.9 (4)	H15B—C15—H15C	109.5
N2—C7—C10	110.5 (3)	C13—C16—H16A	109.5
C8—C7—C10	108.8 (4)	C13—C16—H16B	109.5
C11—C7—C10	111.6 (4)	H16A—C16—H16B	109.5
O2—C8—O3	122.6 (4)	C13—C16—H16C	109.5
O2—C8—C7	125.8 (4)	H16A—C16—H16C	109.5
O3—C8—C7	111.7 (4)	H16B—C16—H16C	109.5
O3—C9—H9A	109.5	C13—C17—H17A	109.5
O3—C9—H9B	109.5	C13—C17—H17B	109.5
H9A—C9—H9B	109.5	H17A—C17—H17B	109.5
O3—C9—H9C	109.5	C13—C17—H17C	109.5
H9A—C9—H9C	109.5	H17A—C17—H17C	109.5
H9B—C9—H9C	109.5	H17B—C17—H17C	109.5
			
C5—N1—C1—C2	−0.2 (5)	C11—C7—C8—O2	114.7 (6)
C5—N1—C1—C6	175.6 (4)	C10—C7—C8—O2	−122.9 (5)
N1—C1—C2—C3	−0.6 (6)	N2—C7—C8—O3	176.8 (4)
C6—C1—C2—C3	−176.2 (4)	C11—C7—C8—O3	−63.8 (5)
C1—C2—C3—C4	1.5 (6)	C10—C7—C8—O3	58.6 (5)
C2—C3—C4—C5	−1.6 (7)	C13—N3—C12—O4	2.3 (8)
C1—N1—C5—C4	0.1 (6)	C13—N3—C12—C5	−178.6 (4)
C1—N1—C5—C12	−178.4 (4)	N1—C5—C12—O4	173.0 (4)
C3—C4—C5—N1	0.8 (7)	C4—C5—C12—O4	−5.5 (7)
C3—C4—C5—C12	179.2 (4)	N1—C5—C12—N3	−6.1 (6)
C7—N2—C6—O1	4.9 (7)	C4—C5—C12—N3	175.4 (4)
C7—N2—C6—C1	−174.3 (3)	C12—N3—C13—C17	70.9 (6)
N1—C1—C6—O1	−178.9 (4)	C12—N3—C13—C14	−52.2 (7)
C2—C1—C6—O1	−3.0 (6)	C12—N3—C13—C16	−168.7 (5)
N1—C1—C6—N2	0.3 (5)	C15—O5—C14—O6	2.2 (10)
C2—C1—C6—N2	176.3 (4)	C15—O5—C14—C13	−172.4 (6)
C6—N2—C7—C8	176.8 (4)	N3—C13—C14—O6	141.2 (6)
C6—N2—C7—C11	58.1 (6)	C17—C13—C14—O6	18.8 (8)
C6—N2—C7—C10	−66.1 (5)	C16—C13—C14—O6	−102.1 (8)
C9—O3—C8—O2	1.1 (8)	N3—C13—C14—O5	−44.3 (6)
C9—O3—C8—C7	179.6 (5)	C17—C13—C14—O5	−166.7 (5)
N2—C7—C8—O2	−4.7 (6)	C16—C13—C14—O5	72.4 (6)

### Hydrogen-bond geometry (Å, º)

**Table:**

*D*—H···*A*	*D*—H	H···*A*	*D*···*A*	*D*—H···*A*
N2—H1*N*2···O2	0.85 (4)	2.08 (4)	2.614 (5)	120 (3)
C3—H3*A*···O6^i^	0.93	2.49	3.204 (6)	134

Symmetry code: (i) −*x*+1/2, *y*, *z*+1/2.
